# Recent Advances in Chemical Biology of Mitochondria Targeting

**DOI:** 10.3389/fchem.2021.683220

**Published:** 2021-05-03

**Authors:** Haiwei Wang, Bin Fang, Bo Peng, Limin Wang, Yufei Xue, Hua Bai, Shenci Lu, Nicolas H. Voelcker, Lin Li, Li Fu, Wei Huang

**Affiliations:** ^1^ Frontiers Science Center for Flexible Electronics, Xi’an Institute of Flexible Electronics (IFE) and Xi’an Institute of Biomedical Materials & Engineering, Northwestern Polytechnical University, Xi’an, China; ^2^ School of Materials Science and Engineering, Northwestern Polytechnical University, Xi'an, China; ^3^ Commonwealth Scientific and Industrial Research Organisation (CSIRO), Clayton, VIC, Australia; ^4^ Drug Delivery, Disposition and Dynamics, Monash Institute of Pharmaceutical Sciences, Monash University, Parkville, VIC, Australia; ^5^ Melbourne Centre for Nanofabrication, Victorian Node of the Australian National Fabrication Facility, Clayton, VIC, Australia; ^6^ Department of Materials Science & Engineering, Monash University, Clayton, VIC, Australia; ^7^ Key Laboratory of Flexible Electronics (KLOFE) & Institute of Advanced Materials (IAM), Nanjing Tech University (NanjingTech), Nanjing, China

**Keywords:** mitochondrial, mitochondrial dysfunction, mitochondrial-targeting molecules, chemical biology, chemical probe, nanomedicine

## Abstract

Mitochondria are vital subcellular organelles that generate most cellular chemical energy, regulate cell metabolism and maintain cell function. Mitochondrial dysfunction is directly linked to numerous diseases including neurodegenerative disorders, diabetes, thyroid squamous disease, cancer and septicemia. Thus, the design of specific mitochondria-targeting molecules and the realization of real-time acquisition of mitochondrial activity are powerful tools in the study and treatment of mitochondria dysfunction in related diseases. Recent advances in mitochondria-targeting agents have led to several important mitochondria chemical probes that offer the opportunity for selective targeting molecules, novel biological applications and therapeutic strategies. This review details the structural and physiological functional characteristics of mitochondria, and comprehensively summarizes and classifies mitochondria-targeting agents. In addition, their pros and cons and their related chemical biological applications are discussed. Finally, the potential biomedical applications of these agents are briefly prospected.

## Introduction

Mitochondria are one of the most important organelles in cells, playing a key role in cell survival. On the one hand, mitochondria synthesize adenosine triphosphate (ATP), the energy required to maintain cell viability by mitochondrial oxidative phosphorylation (OXPHOS); on the other hand, damaged mitochondria produce reactive oxygen species (ROS), cytochrome c and other signals, which initiate apoptosis by activating caspase family proteins (Chan, 2006). Mitochondrial dysfunction is usually characterized as the loss of efficiency of ATP production, which is a characteristic of aging and most all chronic diseases, such as neurodegenerative disorders, cancers and diabetes (Nicolson, 2014). Therefore, mitochondrial targeting therapies might be the key to solving intractable diseases and hold great potential in the treatment of various diseases (Murphy and Hartley, 2018).

A comprehensive analysis of the activity, expression and migration of mitochondrial biomarkers in the process of disease occurrence and development will provide important information for a better understanding of the diseases. Therefore, current attention focuses on the development of sensitive technologies/systems that can identify and monitor mitochondrial functions. In this review, the structure, function and related disease characteristics of mitochondria are introduced. Mitochondrial targeting methods are summarized and classified, such as mitochondria-targeting small molecules, biomolecules and nanomaterials. Moreover, an analysis and discussion of the characteristics of these mitochondria-targeting motifs and related biological applications is provided to the reader. We believe that this review will arouse widespread interest among scientists working on mitochondria and provide chemical biologists with valuable information to further address the challenges.

## The Structure, Function and Related Diseases of Mitochondria

**FIGURE 1:**
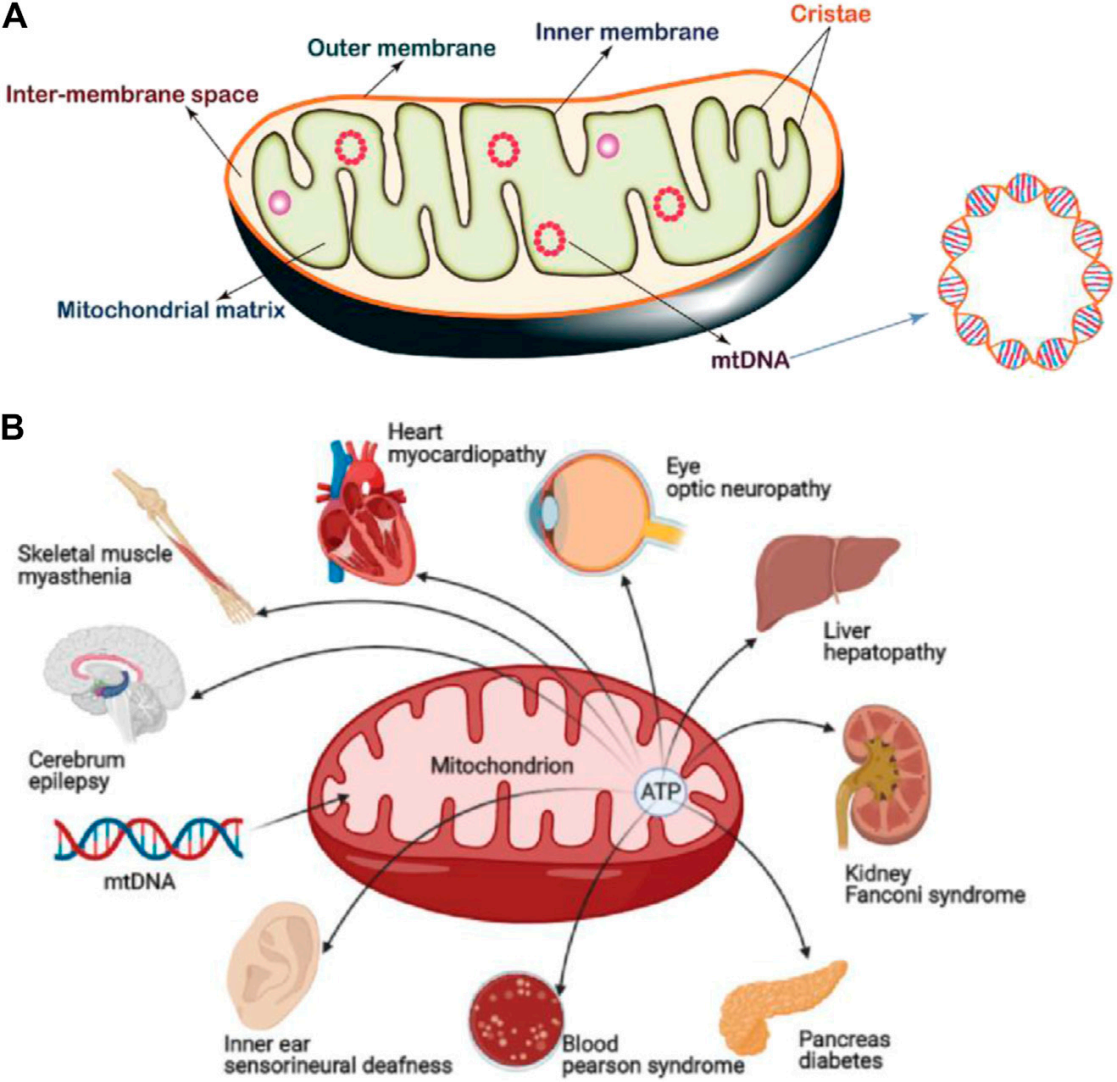
**(A)** Mitochondrial structure, which can be divided by four functional areas: 1) outer mitochondrial membrane (OMM), 2) mitochondrial membrane space (IMS), 3) inner mitochondrial membrane (IMM), and 4) mitochondrial matrix (MM). Mitochondria also possess their own gene information (mtDNA). Image reproduced with permission, from Ref. Samanta et al. (2019). **(B)** Common mitochondrial diseases. 1B was created with BioRender.com.

The structure of mitochondria is very different from other subcellular organelles in the cell. The basic structure of mitochondria can be divided into four functional areas: 1) outer mitochondrial membrane (OMM), 2) mitochondrial membrane space (IMS), 3) inner mitochondrial membrane (IMM), and 4) mitochondrial matrix (MM) (Mannella, 2000). The OMM has a smooth surface morphology and functions as the cell organelle boundary membrane. The specific receptors on the OMM are termed mitochondrial outer membrane complex which selectively recognize and uptake certain substances into the mitochondria. The IMM folds inward to form mitochondrial cristae which results in larger surface area, therefore it is able to carry more biochemical reactions per time unit (Figure 1A). These two membranes define the borders of the extramitochondrial region, inter-membrane space and matrix (Osellame et al., 2012). In addition, the morphology and position of mitochondria in the cell are very important and are strictly regulated by the processes of mitosis, biogenesis and autophagy to ensure the relative stability of the mitochondrial population.

Upon mitochondrial damage, events such as the changes of morphology, membrane potential and permeability to Ca^2+^, reduction of membrane phosphate esters, and oxidative phosphorylation coupling, affect the normal function of the entire cell and lead to the occurrence of diseases. For example, mitochondrial myopathy, cerebral myopathy, Leber's hereditary optic neuropathy etc. are caused by pathological changes after mitochondrial damage (Claudia et al., 2019; Fogle et al., 2019; Winkler et al., 2019).

In addition, a damaged mitochondrial structure and mitochondrial metabolic abnormalities also play important roles in the occurrence and development of many diseases (Viscomi and Zeviani, 2020), (Figure 1B). For example, as a common neurodegenerative disorder, the pathogenesis of Parkinson’s disease (PD) has been strongly linked with mitochondrial dysfunction (Chen et al., 2020). Studies proved that mitochondrial respiratory defects may result in chronic ROS production that leads to the death of dopaminergic neurons. Moreover, disruption of mitochondrial kinetics due to toxic damage or other conditions may also lead to neurodegeneration. In some cases, mitochondrial dysfunction caused by gene mutation is the fundamental cause for the pathogenesis and inheritance of PD (Dauer and Przedborski, 2003; Thomas and Beal, 2007; Bose and Beal, 2016). Therefore, diseases where the progression can be correlated with or compensated for mitochondrial function are collectively referred as mitochondrial diseases. At present, in addition to neurodegenerative disorders (Baldassarro et al., 2019), there are numerous diseases related to abnormal mitochondrial structure and function, including mental diseases (Ashwini et al., 2015), tumor (Schubert et al., 2020), aging (Bornstein et al., 2020), cardiovascular disease (Veloso et al., 2019), diabetes (Szendroedi et al., 2012), etc. Therapeutics which target mitochondria also result in positive responses in some cases (Jeena et al., 2019). Although these diseases appear in different tissue sites and show different symptoms, mitochondrial dysfunction is the common feature, mainly manifested as insufficient production capacity due to impaired oxidative phosphorylation, increased ROS, and abnormal apoptosis signals (Lesnefsky and Hoppel, 2006).

In recent years, research on rectifying the structure and function of mitochondria has been carried out rapidly. For example, fixing mitochondrial DNA mutations through genome editing technology was reported to have a certain curative effect on angiocardiopathy (Gammage et al., 2018). In addition, diagnostic and therapeutic agents targeting mitochondria, such as a substance called Gboxin, an inhibitor of oxidative phosphorylation, can produce specific inhibition of diabetes or glioma (Shi et al., 2019). Although great efforts have been put into the treatment of mitochondria-related diseases (Slone and Huang, 2020), in most cases, the structure and function of mitochondria have been irreversibly damaged which result in limited therapeutic effects. Alternatively, a newly emerged approach, mitochondrial replacement therapy (MRT), which supplements the cells with healthy mitochondria is a promising approach to fundamentally treat mitochondria-related diseases (Schumacker et al., 2014) (Mccully et al., 2016). In addition, combination therapies such as phototherapy and small molecule anticancer drugs have been developed as alternatives to synergistic treatment (Xie et al., 2020).

## Mitochondria-Targeting Agents

For mitochondria therapies and biological studies, the selectivity or even specificity of agents targeting the mitochondrial structures is essential to the design of corresponding therapeutics and probes. This selectivity will result in less off-target toxicity and also lower the effective concentration of drugs/probes, therefore enhancing the corresponding bioavailability (Jhaveri and Torchilin, 2016; Yao et al., 2021). Substantial progress in the understanding of mitochondria provides critical information that paves the way for the design of mitochondrial targeting methods and agents.

### Mitochondria-Targeting Small Molecules

Due to the presence of a proton pump (PP) in the IMM, the protons in the MM are pumped into the IMS, resulting in positive charges in the IMS. MM carries negative charges, which form the transmembrane potential (MTP) across the IMM, which is also known as mitochondrial membrane potential (Zorova et al., 2018). There is usually a negative potential difference (∼180–200 mV) on both sides of the IMM, which is the mitochondrial membrane potential (∆Ψm) (Smith et al., 2012). Changes in mitochondrial membrane potential or even minor changes will greatly affect the function of mitochondria.

**FIGURE 2:**
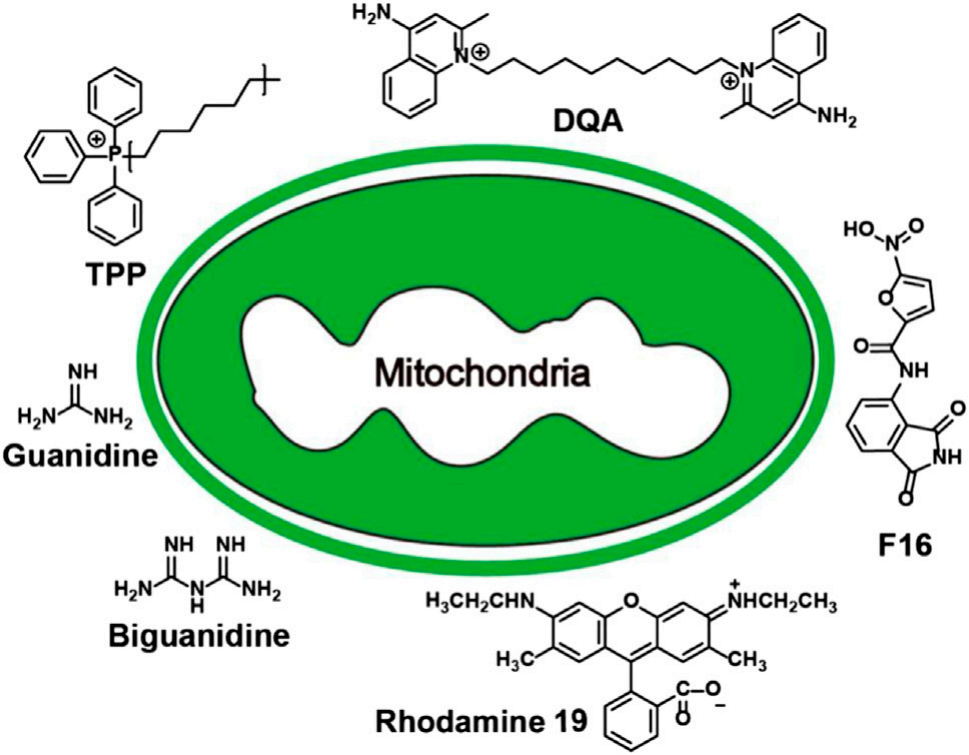
Summary of mitochondria-targeting small molecules.

Existing mitochondria-targeting small molecules include triphenylphosphonium, dequalinium, (E)-4-(1H-Indol-3-ylvinyl)-N-methylpyridineiodide, tetramethylrhodamine ethyl ester (TMRE), guanidine salt, tetramethylrhodamine methyl ester (TMRM), rhodamine 19, rhodamine 123 and tetrachlorotetraethyl benzimidazole carbocyanine iodine Compounds (5,5′,6,6′-tetrachloro-1,1′,3,3′-tetraethyl-imidacarbocyanine, JC-1) (Chazotte, 2011; Perelman et al., 2012; Cunha-Oliveira et al., 2018) (Figure 2). Most of them are delocalization lipophilic cations (DLCs). The lipid solubility of these molecules enables them to cross the cell membrane and mitochondrial membrane, and the positive charge enables them to enter MM under the action of the mitochondrial membrane potential, endowing them with mitochondrial targeting ability (Murphy, 2008). In addition, because the MTP of normal cell is lower than that of cancer cells and transformed cells, DLCs can preferentially concentrate in the pathological cells (Kalyanaraman et al., 2018). Most of these small molecules were discovered decades ago. Therefore, the use of small molecule-based mitochondria-targeting molecules for various biological applications is relatively mature.

#### Triphenylphosphonium (TPP^+^)

**FIGURE 3:**
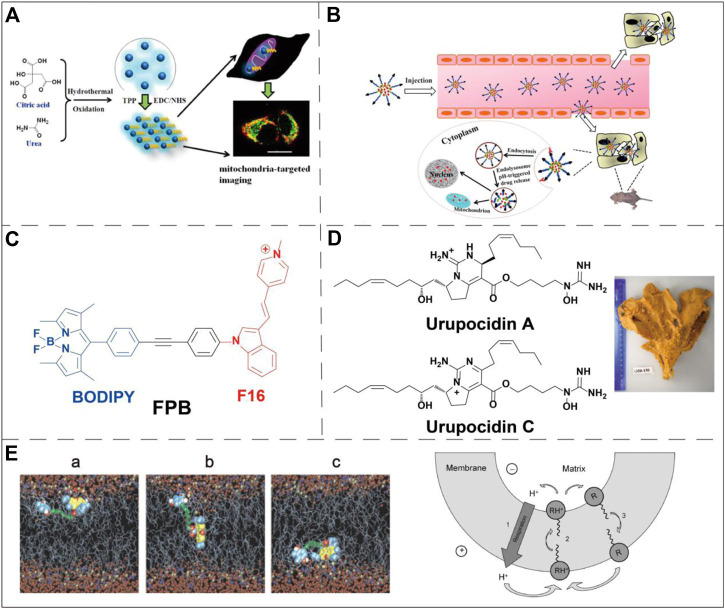
Examples of mitochondria-targeting small molecules applications. **(A)** TPP^+^ was conjugated to fluorescent carbon dots to selectively monitor mitochondria via one- or two-phono live cell image. Image reproduced with permission, from Wang et al. (2014). **(B)** Schematic diagram for the doxorubicin delivery in breast cancer cells using a pH-responsive DQA-Dox micelle system. Image reproduced with permission, from Ref Song et al. (2015). **(C)** Chemical structure of a conjugate molecule of F16 and BODIPY, F16 was used as a guiding ligand for mitochondria. He et al. (2015). **(D)** Marine alkaloid sourced and guanidine-containing complex capable of targeting mitochondria. Image reproduced with permission, from Ref Dyshlovoy et al. (2020). **(E)** Snapshot of the permeation of Rh 19 derivative through bilayer lipid membrane (left panel) and schematic diagram of its mechanism (right panel, where R stands for a deprotonated neutral form, and RH^+^ stands for a singly protonated cationic form). Image reproduced with permission, from Ref Antonenko et al. (2011).

The most successful case of using lipophilic cations for mitochondrial targeting is the discovery of triphenylphosphonium (TPP^+^) (Liberman et al., 1969; Wang et al., 2020). The review written by Kalyanaraman et al. comprehensively summarizes the structures and synthesis of TPP^+^-based compounds and their applications in biology (Zielonka et al., 2017). Here we will briefly highlight several biological applications using TPP^+^ for mitochondrial targeting. TPP^+^ contains three benzene rings that enable incorporation into many functional molecules and form a delocalized positive charge that can pass through the mitochondrial double-layer hydrophobic membrane. Many biologically active molecules have been conjugated to TPP^+^ to realize mitochondrial targeting. For example, the antioxidant vitamin E linked to TPP^+^ can more effectively protect mitochondria from oxidation. Chemically linking doxorubicin (Dox) and TPP^+^ showed a good mitochondria-targeting effect, and induced tumor cell apoptosis through the mitochondrial pathway, overcoming tumor cell drug resistance (Kalinovich et al., 2016). The chemical combination of TPP^+^ with nano-formulations also has the effect of mitochondrial targeting. For example, paclitaxel-loaded liposomes prepared using TPP^+^-modified polyethylene glycol-phosphatidylethanolamine (PEG-PE) have shown to be effective in targeting mitochondria in cancer cells (Biswas et al., 2012a). Similar designs include TPP^+^-modified polyamidoamine (PAMAM), coumarin-iron oxide (CIO), gold nanoparticles (AuNPs) and polyglycolic acid-polyethylene glycol (PLGA-PEG) nanoparticles, etc (Biswas et al., 2012b; Jung et al., 2015; Marrache and Dhar, 2015; Pathak et al., 2015; Agrawal et al., 2017; Wang et al., 2017). All these TPP^+^-modified nanovehicles exhibit mitochondrial targeting ability and excellent anti-tumor effect *in vivo* and *in vitro*. In addition, TPP^+^ has been widely used in the design of fluorogenic probes for the detection of mitochondrial biomarkers (Wang et al., 2014; Fu et al., 2021) (Figure 3A). By conjugating TPP^+^, a NIR reporter, and a sulfenic acid-reactive group, Gao et al. synthesized a novel NIR probe DATC, which is able to visualize endogenous protein sulfenic acids expressed in the mitochondria (Gao et al., 2020).

#### Dequalinium (DQA)

Dequalinium (DQA) is a lipophilic compound discovered by Weiss et al*.* (1987). DQA consists of two cationic quinoline groups connected by a 10-carbon alkyl chain. It is a delocalized lipophilic cationic molecule with a mitochondrial targeting effect. Weiss et al. found that this compound inhibited the proliferation of multiple cancer cell lines both *in vitro* and *in vivo*. Studies have shown that DQA can induce ROS production by inhibiting ATP synthesis, which further leads to the expression of cytochrome c and the decline of mitochondrial membrane potential. This pathway finally activates the caspase-3/9 dependent endogenous apoptosis pathway (Sancho et al., 2007). Therefore, the cytotoxicity of DQA limits its application in many biological studies, such as fluorescent probes or cell biology research. However, the cytotoxicity also makes DQA an excellent drug delivery ligand in the field of anti-cancer therapy. Studies have conjugated DQA chloride with Dox (DQA-Dox) to achieve targeted delivery of Dox to mitochondria. The drug conjugate DQA-Dox was found mainly accumulating in the mitochondria of MCF-7/ADR cells, and exhibited high cancer cell cytotoxicity (Song et al., 2015) (Figure 3B).

#### (E)-4-(1H-Indol-3-ylvinyl)-N-Methylpyridineiodide (F16)

As a delocalized lipophilic cationic molecule, (E)-4-(1H-Indol-3-ylvinyl)-N-methylpyridineiodide (F16) exhibits a mitochondrial targeting effect. It was initially discovered via cell-based high-throughput screening. This small molecule can selectively inhibit the proliferation of a variety of cancer cell lines (Rathinavelu et al., 2017). Similar to DQA, F16 itself is cytotoxic, its accumulation in mitochondria can cause the depolarization of mitochondrial membranes, destroy the mitochondrial structure, leading to the opening of the mitochondrial permeability transformation channel. The opening of the pores subsequently causes the production of cytochrome c and promotes cell apoptosis (Spivak et al., 2021). By combining F16 with the widely used boron-dipyrromethene (BODIPY) fluorescent dye through a phenylethynyl linker, a dual-functional mitochondria-targeting molecule was prepared (Figure 3C). This conjugate possesses anti-cancer activity, optical properties suitable for bioimaging and cancer cell specificity. Cell-based viability assays showed that the conjugate’s IC_50_ against SGC-7901 cells is very close to that of its BODIPY-free precursor, F16. This result suggests that the installation of BODIPY (without obvious toxicity in free form) does not interfere the cytotoxicity of the original drug F16 (He et al., 2015).

#### Guanidine/Biguanidine

Both guanidine and biguanide are delocalized lipophilic cationic molecules, which have a delocalized positive charge and therefore exhibit stronger lipophilicity than groups with a localized charge. Some studies have found that a new marine guarantin alkaloid can selectively kill prostate cancer cells and that the cytotoxic effect is related to mitochondrial targeting (Dyshlovoy et al., 2020) (Figure 3D). This study confirmed for the first time that mitochondrial targeting is the central mechanism of the anti-cancer effect of these molecules and their derivatives. At the same time, it was proposed that isolated alkaloids could be used to treat mitochondrial membrane infiltration, and then release cytotoxic mitochondrial proteins into cell cytoplasm, upregulate ROS, and finally promote apoptosis of prostate cancer cells. Similar to DQA and F16, the cytotoxicity of guanidine and biguanide limit their applications in certain types of biological studies.

#### Rhodamine

Rhodamine 123 (Rh123) is the most common member of the rhodamine-based targeting agents. Johnson and his colleagues verified the localization of Rh123 in the mitochondria of living cells in 1980 (Johnson et al., 1980). Due to the lipophilic and cationic properties, Rh123 is able to penetrate the IMM driven by the mitochondrial membrane potential and accumulate in the MM. As a fluorescent dye, Rh123 has been widely used to measure the mitochondrial membrane potential (Pan et al., 2020). Notably, Rh 123 exhibits selective anticancer activity in *in vivo* tumor models. The combination of 2-deoxyglucose or methylglyoxal bisamidinylhydrazone with Rh123 may also further enhance this selective cytotoxicity (Kageyama et al., 2005). However, in relevant clinical experiments, the maximum tolerated dose of Rh123 is low, and excessive Rh123 exhibited severe cytotoxicity (Jones et al., 2005). Rhodamine 19 (Rh19), the successor of Rh123, exhibits considerable mitochondrial targeting activity, and more recently has replaced TPP^+^ for the design of drug conjugate showing excellent mitochondrial targeting ability and anti-cancer activity (Antonenko et al., 2011) (Figure 3E). Compared with TPP^+^, intrinsic fluorescence of Rh19 enables live-time monitoring mitochondrial via confocal microscopy. Rh19 has also been utilized to study the expression behavior of related active substances in mitochondria by means of super-resolution micro-imaging (Miljanic et al., 2002; Khailova et al., 2014); In addition, Rh19 is less cytotoxic than TPP^+^ which further extend its applications in various biological research (Rogov et al., 2016).

### Transition Metal Complex Targeting

The use of metal complexes in biological applications has seen remarkable advances in recent decades (Allardyce et al., 2005; Coogan and Fernández-Moreira, 2014). These modularly prepared metal complexes usually contain easily modified organic ligands (Lo and Kam-Wing, 2015; Wang et al., 2016). In recent years, metal complexes that specifically target organelles have been conjugated to anticancer drugs in order to improve the therapeutic effect of the drugs (Ma et al., 2014).

**FIGURE 4:**
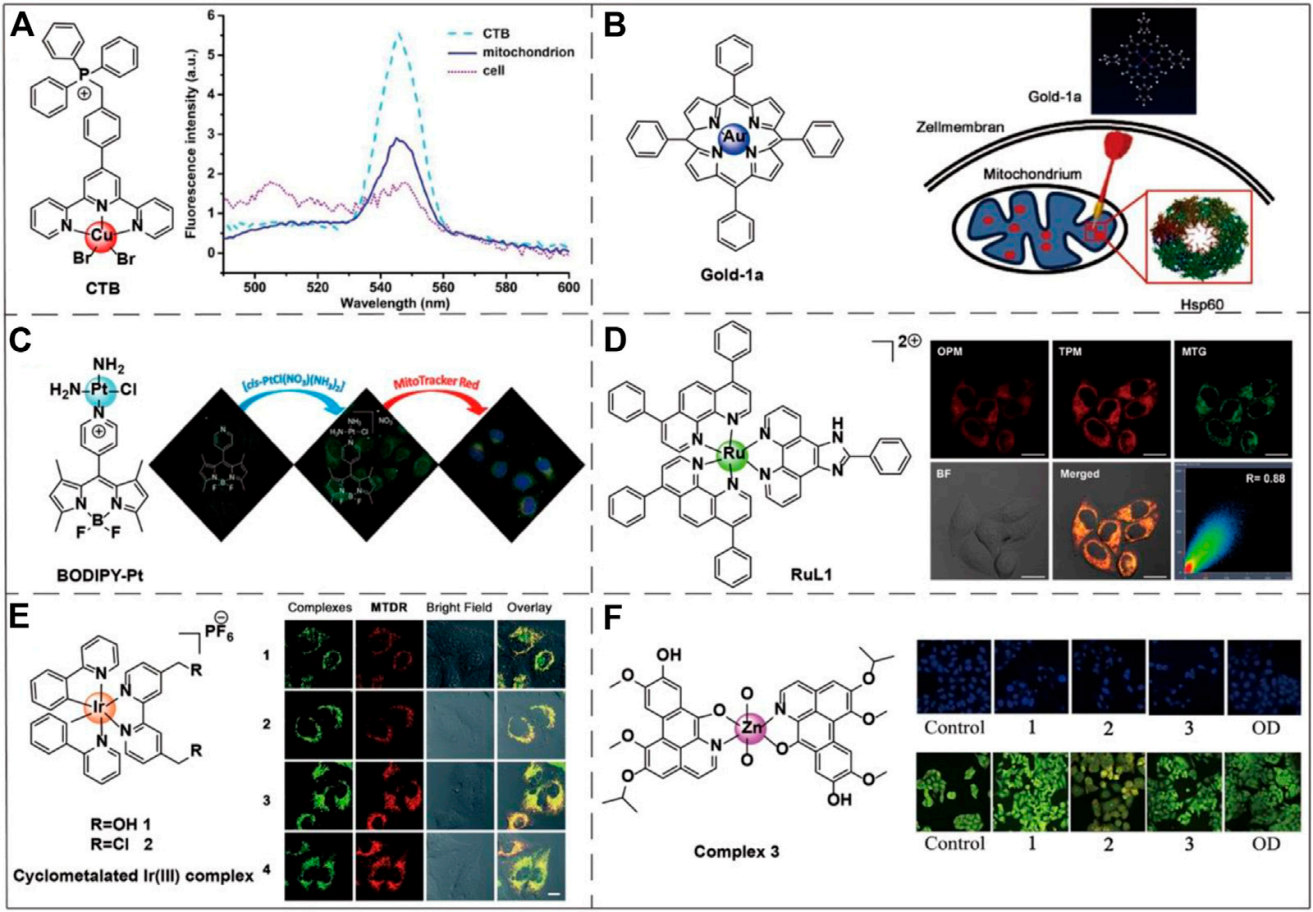
Chemical structures of six transition metal complexes and the corresponding applications. **(A)** The chemical structure of CTB and its fluorescence spectra in different biological environments. Image reproduced with permission, from Zhou et al. (2014). **(B)** The chemical structure of gold-1a and its effective targeting to the mitochondrial chaperone Hsp60. Image reproduced with permission, from Ref Hu et al. (2016). **(C)** The chemical structure of BODIPY-Pt is also sensitive to mitochondrial membrane potential. On the right side of the figure is the fluorescence diagram of BODIPY-Pt under MitoTracker™ Red. Image reproduced with permission, from Ref Sun et al. (2015). **(D)** RuL1 is a Ru(II) polyamide complex-based photodynamic anticancer drug that targets mitochondria. On the right side of the figure are the one-photon (OPM) and two-photon (TPM) fluorescence imaging of the Ru(II) metal complex colocalized with the MitoTracker™ Green (MTG) in HeLa cells. Image reproduced with permission, from Ref Liu et al. (2015). **(E)** An example of Ir(III) transition metal complex structure capable of accumulating within mitochondria. The right-hand side of the figure shows the fluorescence of the Ir(III) metal complex in A549 cells nicely colocalized with the MitoTracker™ Deep Red staining. Image reproduced with permission, from Ref Cao et al. (2017). **(F)** An example of a Zn(II) transition metal complex structure capable of inducing mitochondrial apoptosis. The right side of the figure shows the DAPI nuclei staining (blue color) and AO/EB viability staining (green color) of HepG2 cells apoptosis caused by three transition metal complexes via a caspase-dependent mitochondrion pathway. 1, 2, and 3, respectively, represent Co(II), Ni(II), and Zn(II) complexes. The oxoaporphine derivative (OD) has good anti-tumor activity and was used as the positive control. Image reproduced with permission, from Qin et al. (2017).

Zhou et al. designed a copper complex CTB ([Cu(ttpy-tpp)Br_2_]Br (ttpy-tpp = 4′-*p-*Tolyl-(2,2’:6′,2″-terpyridyl)triphenylphosphonium bromide) which could target mitochondria in drug-resistant tumor cells and overcome the resistance to cisplatin (Zhou et al., 2014) (Figure 4A). Hu et al. demonstrated that gold (III) meso-tetraphenylporphyrin (gold-1a) had excellent mitochondrial targeting ability and potential anticancer activity via targeting heat-shock protein 60 (Hsp60) (Hu et al., 2016) (Figure 4B). Sun et al. found that a metal complex named BODIPY-pt which was composed by the combination of the fluorescent dye BODIPY and platinum (PtIV) could be selectively ingested by mitochondria. BODIPY-pt also exhibited excellent anti-proliferative activities against human cervical cancer (HeLa) and breast cancer (MCF-7) cell-lines (Sun et al., 2015) (Figure 4C). Recent studies have shown that ruthenium (RuII) (Figure 4D) and iridium (IrIII) (Figure 4E) complexes can target mitochondria (Liu et al., 2015; Cao et al., 2017; Yao et al., 2021). Especially in the treatment of cancer by photothermal therapy (PTT), the complexes with these two transition metals as the metal center can effectively target the mitochondria of cancer cells and enhance the therapeutic effect (Liu et al., 2015). Qin et al. designed three transition metal complexes with cobalt, nickel, and zinc as metal centers to conduct a comparative anti-cancer activity test. The experiments showed that all three transition metal complexes exhibited excellent anti-tumor activity and have great potential as anti-cancer drugs (Qin et al., 2017) (Figure 4F).

### Mitochondria-Targeting Bioactive Molecule

Peptides have been widely used in the biological field due to their excellent selectivity, high activity and the mature solid-phase peptide synthesis (Boyle and Woolfson, 2011). For mitochondria-targeting peptides, various short peptides and polypeptides have been discovered which are proposed as an alternative to lipophilic cations for mitochondria-targeting molecules. These peptides usually carry hydrophobic (such as phenylalanine, tyrosine, isoleucine) and positively charged (such as arginine, lysine) amino acids. The corresponding mechanism for mitochondrial targeting is mainly based the targeting of mitochondrial membrane potential or the mitochondrial transmembrane proteins. Although there are numerous studies on mitochondria-targeting peptides, there is still plenty of scope for this approach and potential to be further explored.

#### Mitochondria-Penetrating Peptides (MPPs)

**FIGURE 5:**
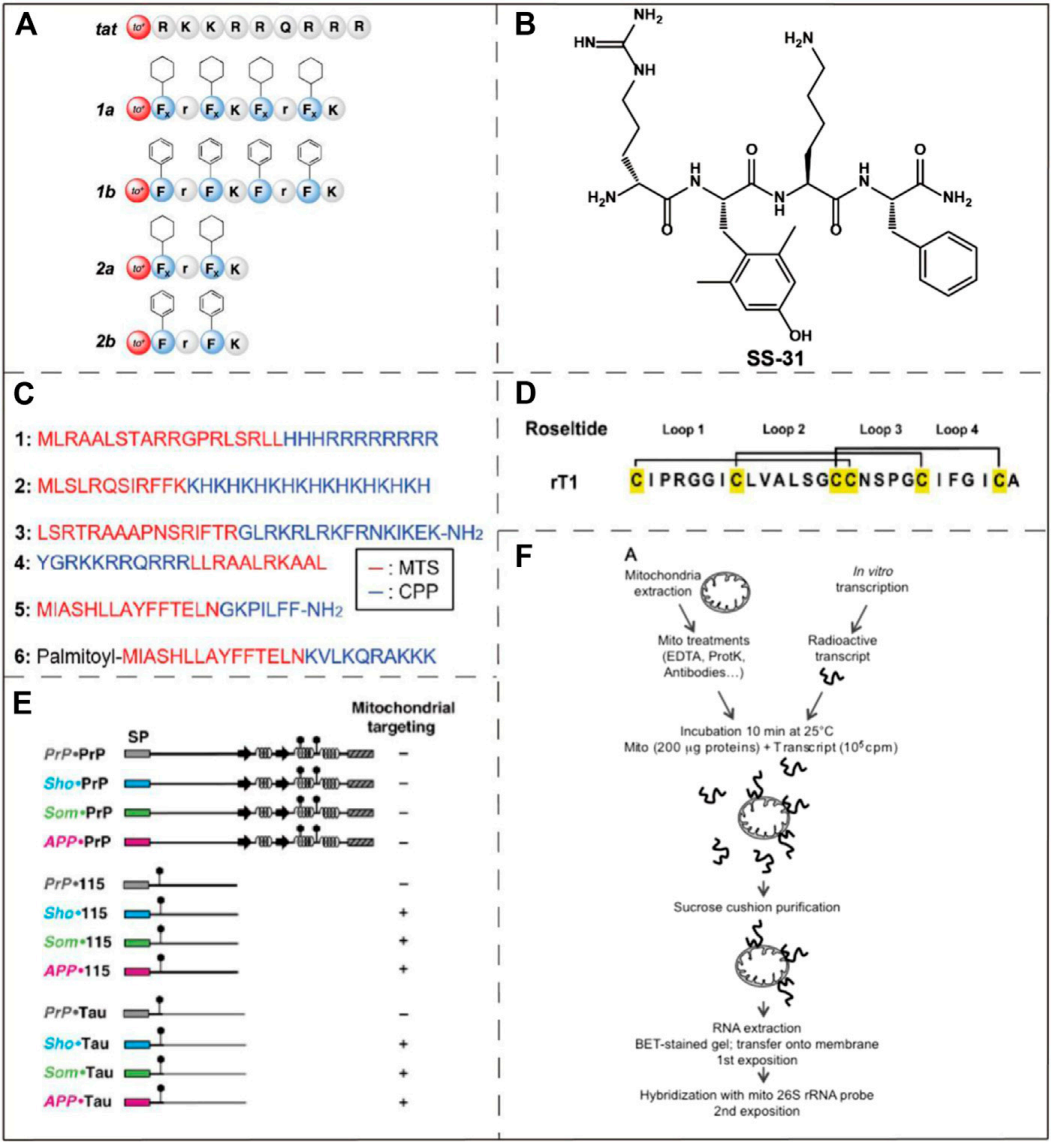
Examples of mitochondria-targeting bioactive peptides and nucleic acids. **(A)** The structure of an array of MPPs which were designed by Yousif et al. Image reproduced with permission, from Ref Yousif et al. (2010). **(B)** The chemical structure of SS-31. Image reproduced with permission, from Ref Birk et al. (2013). **(C)** Amino acid sequences of six MTSs. Image reproduced with permission, from Ref Kim et al. (2020). **(D)** The structure of roseltide rT1. Image reproduced with permission, from Ref Kam et al. (2019). **(E)** Identification of mitochondrial targeting states by different ER signal peptides. Image reproduced with permission, from Ref Pfeiffer et al. (2013). **(F)** Naked mRNA can bind to isolated mitochondria. Reproduced, with permission, from Ref Michaud et al. (2014).

Mitochondria-penetrating peptides (MPPs) are a type of widely utilized mitochondria-targeting molecules that have been discovered in 2008. Horton et al. gave a comprehensive introduction to the discovery process and the synthetic methods of MPPs (Horton et al., 2008). Wu et al. summarized recent advances in the applications of MPPs in cancer therapy via mitochondrial targeting (Wu et al., 2018). MPPs have excellent cell permeability and mitochondrial targeting because they have alternating cationic and hydrophobic residues. At the same time, Horton et al. consider that the uptake of MPPs by cells seems to be independent of the endocytosis pathway, excluding endosome/lysosome isolation, which also increases the chance of MPP reaching mitochondria. Yousif et al. designed an array of MPPs (Figure 5A), which is a short peptide library that can penetrate both cell membranes and mitochondrial membranes (Yousif et al., 2010). Chuah et al. used lysine-histidine (KH) peptide as a cell-penetrating peptide (CPP). KH peptide was coupled to two different mitochondria-targeting signals molecules (a 12-mer peptide from cytochrome c oxidase and a 32-mer peptide from ornithine transaminase). The resulting molecule encapsulated plasmid DNA by self-assembly and successfully realized mitochondrial transfection (Chuah et al., 2016). However, the use of mitochondrial membrane potentials to target mitochondria has not been fully explored yet (Wu et al., 2018).

#### Szeto-Schiller (SS) Peptide

Szeto-Schiller (SS) peptides named for the researchers who discovered them (Zhao et al., 2004) constitute another type of mitochondria-targeting peptide. These peptides were initially developed as antioxidants, but significant accumulation in the IMM was observed. SS peptides have antioxidant activity due to its 2,4-dimethyltyrosine (Dmt) residues. In addition to reducing mitochondrial ROS, they also inhibit the production of cytochrome c. With the continuous optimizations on SS peptides, several prominent derivatives, for instance SS-31, have been reported. As one of the most potent SS-peptides, SS-31 has been widely explored in many disease models such as neurodegenerative disorders, heart failure and ischemia-reperfusion injury (Szeto and Schiller, 2011). SS-31 is currently in phase II clinical trials, as a treatment for ischemia-reperfusion and microvascular injury (Wang et al., 2017). Although it has been proved that SS-31 is selectively localized to the IMM by interacting with cardiolipin, the exact mechanism of its mitochondrial targeting ability is still unclear (Birk et al., 2013) (Figure 5B).

#### Mitochondria-Targeting Sequences (MTSs)

Mitochondria-targeting sequences (MTSs) are usually composed of 20–40 amino acids, which usually bind to specific receptors on mitochondrial membrane (Neupert, 2015) (Figure 5C). The specific receptors commonly include a translation enzyme of the OMM (e.g. translocator of the OMM) and translation of inner membrane complex (e.g. translocator of the IMM). They play a role in the introduction of MTSs into the mitochondria, mainly powered by ATP or mitochondrial transmembrane potential. MTSs have some disadvantages which are its large structure, low solubility and insufficient permeability across the cell membrane (Yousif et al., 2009). Despite these shortcomings, the excellent selectivity and low toxicity of MTSs make them an appropriate choice in certain biological applications. Mossalam et al. fused tumor suppressor p53 to several MTSs to promote mitochondrial-induced rapid apoptosis (Mossalam et al., 2012). These MTSs are derived from Bcl-XL, the adventitia translocation enzyme (TOM 20), cytochrome C oxidase subunit VIII, and ornithine transcarbamylase, respectively. The MTSs applied that study target either the OMM or IMM by specifically recognizing and binding to the corresponding proteins. The result demonstrated that the interactions between p53 and mitochondria membrane proteins allows tuning of the apoptosis induction efficiency. The assisted p53-Bcl-XL interaction induced the greatest increase in programmed cell death. In the genetic study of Drosophila, Li et al. revealed the important role played by the mitochondrial protease YME1L in the clearance of poly (GR), and also found that poly (GR) could be potentially used as a mitochondria-targeting sequence (Li et al., 2020).

#### Cysteine-Rich Peptides (CRPs)

Cysteine-rich peptides (CRPs) are natural peptides that can be extracted from plants and have broad development opportunities (Kim et al., 2020). CRPs contain multiple cysteine residues and form special disulfide bonds, the excellent rigidity and tightness of which provide metabolic stability under physiological conditions (Figure 5D). Tam et al. confirmed a novel class of CRPs, roseltides (rT1-rT8), are human neutrophil elastase inhibitors (Loo et al., 2016). Among them, roseltide rT1 was originally developed as an inhibitor of a protease in humans, but it was found to have structural features that target mitochondria. Roseltide rT1 contains a positively charged loop1 and a hydrophobic loop2, which can be specifically recognized by translocator of the OMM and transport roseltide rT1 to the MM (Kam et al., 2019). The sequences of roseltide rT1 and MTSs have the common feature of amphiphilic helix structure. Roseltide rT1 has several advantages, such as resistance to proteolysis and rapid mitochondrial localization.

#### Endoplasmic Reticulum (ER) Signal Peptide

In eukaryotic cells, protein transport and targeting specific organelles is critically important to cell function and homeostasis. The ability to mobilize proteins to the endoplasmic reticulum (ER) and mitochondria mainly depends on the structure of the N-terminal signal peptides of the proteins. Pfeiffer et al. conducted a study and unexpectedly found that ER signal peptides from three special proteins can specifically target mitochondria (Pfeiffer et al., 2013) (Figure 5E). ER targeting occurs during protein co-translation, and mitochondrial targeting occurs after protein translation. The ER signal peptides can mediate ER co-translation and introduction. Therefore, when the ER signal peptides are fused with the N-terminal of the foreign polypeptides, the ER signal peptides have the ability to shuttle the foreign polypeptide into the ER. However, when the attached polypeptides are intrinsically disordered domain, the ER signaling peptide will introduce them into the mitochondria. Meanwhile, experimental studies have shown that the greater the effect of ER signal peptides on mitochondrial membrane potential, the lower its efficiency of introducing into ER. It is thus concluded that the targeting of ER signal peptides to mitochondria is inversely proportional to the ER targeting efficiency of heterologous polypeptide. At the same time, results also indicated that ER signal peptides can be used to target the MM and can affect the function of mitochondria. However, the exact mechanism of these ER signal peptides targeting mitochondria has not been fully illustrated.

#### mRNA

Among the proteins in the mitochondria, only a small fraction of the proteins produced are encoded by the mitochondrial genome, most of them are encoded by the nucleus and then imported into the mitochondria. These foreign proteins are translated by the free cytoplasmic multimers and finally transported to the mitochondria. It has also been verified that a large number of cytoplasmic genes encoding mitochondrial proteins are found on the surface of mitochondria in both plants and animals (Marc et al., 2002; Michaud et al., 2010; Matsumoto et al., 2012). Michaud et al. conducted an *in vitro* binding assay using isolated plant mitochondria to naked mRNA, and the results showed that naked mRNA can specifically bind to isolated mitochondria *in vitro*, but required the participation of the mitochondrial outer membrane complex (Michaud et al., 2014) (Figure 5F). Therefore, some mRNAs may allow specifical targeting of mitochondria, but there is no clarity in relation to the relevant mechanisms.

### Mitochondria-Targeting Nanomaterials

Mitochondria-targeting molecule-drug conjugates usually have disadvantages such as poor water solubility and cytotoxicity which limit their further applications in the clinic. One solution is to deliver therapeutic reagents to the tumor mitochondria using a nanoscale drug delivery system (DSS). Mitochondria-targeting nanomaterials generally have two ways to achieve mitochondrial targeting: 1) mitochondrial targeting is achieved by linking with targeted cationic molecules or biophilic peptide molecules, 2) a small number of nanomaterials of which the intrinsic physiochemical properties enable mitochondrial targeting. Commonly used mitochondria-targeting nanomaterials include liposomes, micelles, dendrimers, carbon nanoparticles, and metal nanoparticles or nanoclusters. Our recent review comprehensively summarized the bio-applications of the mitochondria-targeting DDSs (Yao et al., 2021).

#### Polymeric/Polymer-Coated Nanoparticles and Micelles

**FIGURE 6:**
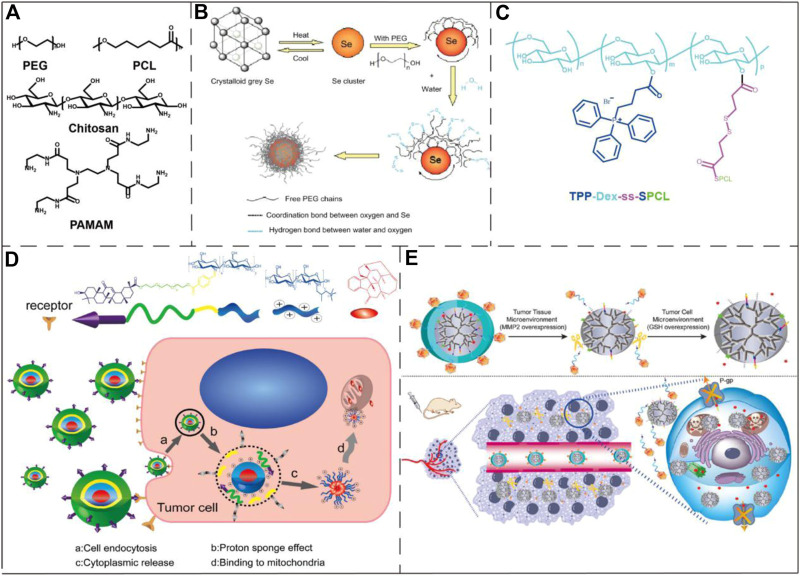
Examples of polymeric/polymer-coated nanoparticles and micelles in the application of drug delivery to mitochondria. **(A)** The chemical structures of PEG, PCL, chitosan, and PAMAM. **(B)** Schematic diagram of a PEG-coated selenium cluster. Image reproduced with permission, from Ref Zheng et al. (2012). **(C)** The chemical structure of a mitochondria-targeting co-polymer with TPP^+^ side chains. Image reproduced with permission, from Ref Tan et al. (2019). **(D)** Schematic illustration of the chitosan nanoparticle mitochondrial targeting process. The nanoparticles were first internalized (a) and then escaped from the endosome into the cytosol (b–c). Lastly, the nanoparticles reached to mitochondria via the positively charged quaternary amine groups and induced cell apoptosis. Image reproduced with permission, from Chen et al. (2015). **(E)** The upper part of the figure represented that the conjugate consisting of PAMAM and enzymatic detachable glucose-PEG was first exfoliated by the outer PEG layer after the action of matrix metalloproteinase 2, and then glutathione stimulated the release of PTX. The lower part of the figure shows the action process of the conjugate in the tumor-bearing mouse model. Image reproduced with permission, from Ref Ma et al. (2018).

Polymer nanoparticles are assembled from amphiphilic polymers which have the advantages of high biocompatibility, low toxicity, high drug loading capacity, small size, easy modification and good aqueous solubility (Mahapatro and Singh, 2011). Some polymeric nanoparticles and micelles have been used in mitochondrial targeting, including polyethylene glycol (PEG), chitosan, dendrimers, polycaprolactone (PCL), hyaluronic acid and some micelles formed by small amphiphilic molecules, etc (Figure 6A). Zheng et al. reported the preparation of ultrasmall selenium nanoclusters with PEG (PEG-SeNCs) (Figure 6B). PEG-SeNCs exhibited stronger growth inhibition and induced concentration-dependent apoptosis to the drug-resistant hepatocellular carcinoma (R-HepG2) cells. Further molecular investigation revealed that the anti-cancer effect was due to the depletion of mitochondrial membrane potential and generation of superoxide anions (Zheng et al., 2012). Tan et al. created a PCL-based mitochondria-targeting DDS, by using TPP^+^ as mitochondria-targeting molecule. The resulting nanoparticle was applied in the treatment of metastatic breast cancer (Tan et al., 2019) (Figure 6C). Chen et al. designed a multifunctional chitosan nanoparticle which was able to achieve efficient intracellular transport and mitochondrial positioning and improve the anti-tumor efficacy (Figure 6D). The mitochondrial targeting was realized through the interaction of the positively charged nanoparticles and cancer cell mitochondria with the higher negative potential (Chen et al., 2015). Ma et al. constructed a mitochondria-targeting conjugate consisting of PAMAM and enzymatic detachable glucose-PEG that transported paclitaxel (PTX) into the mitochondria (Figure 6E). Their research results show that this conjugate can well target mitochondria and act on tumor cells, and can be used to make up for the multi-drug resistance problem of PTX (Ma et al., 2018).

#### DQAsomes and DQA-Liposomes

**FIGURE 7:**
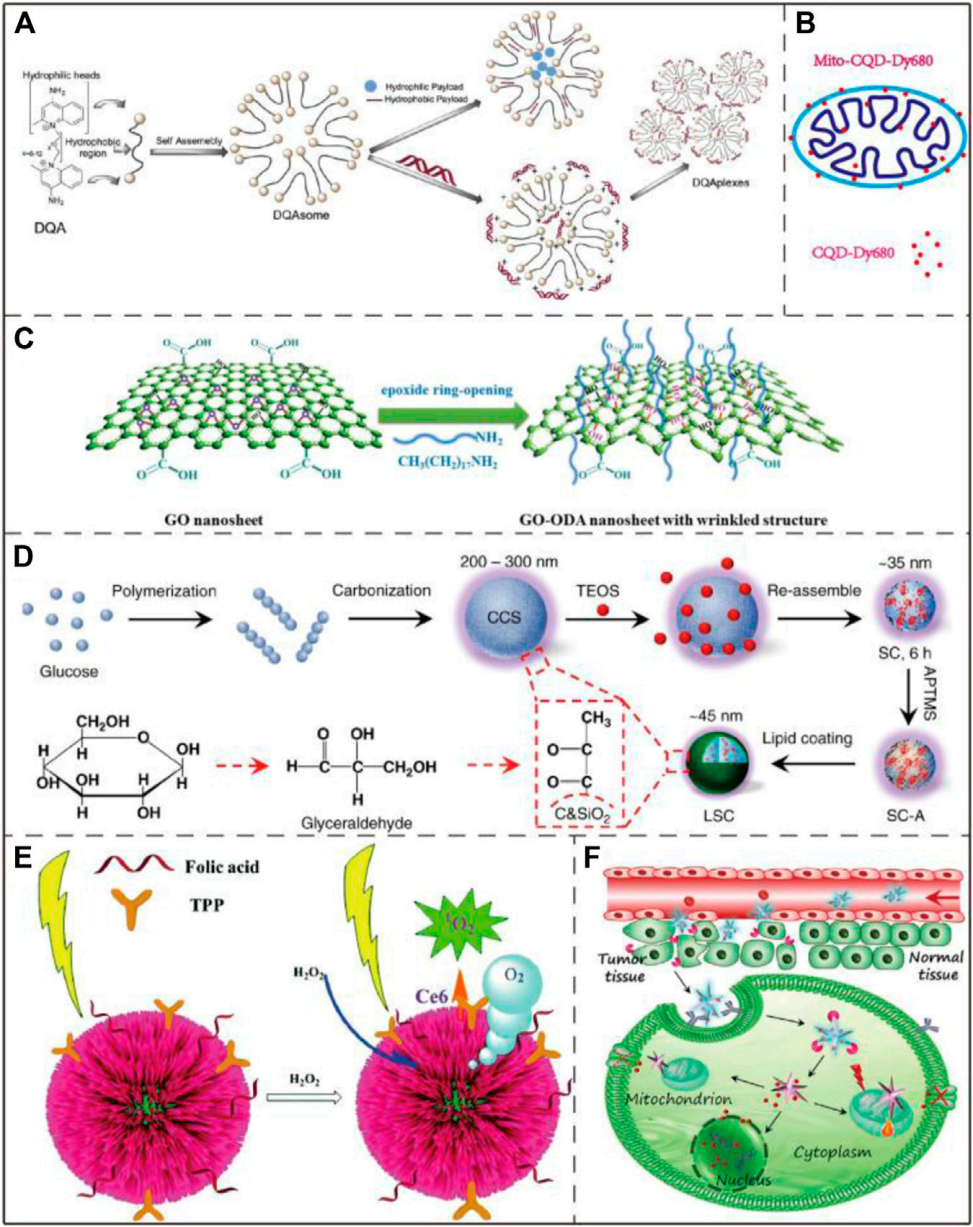
Application examples of DQAsomes and four different types of inorganic nanoparticles. **(A)** Chemical structure of DQA and its self-assembly into liposome-like vesicles. Image reproduced with permission, from Ref Pathak et al. (2015). **(B)** A mitochondrial transport system based on CQDs. Image reproduced with permission, from Ref Zheng et al. (2018). **(C)** Schematic diagram of GO functionalization process. Image reproduced with permission, from Ref Zhang et al. (2020). **(D)** Schematic diagram of LSC-based nanoparticle fabrication. CCS, colloidal carbon sphere; TEOS, tetraethyl orthosilicate; SC, silica-carbon; APTMS (3-Aminopropyl) trimethoxysilane. Image reproduced with permission, from Ref Wang et al. (2018). **(E)** Schematic diagram of three-dimensional structure of Au@Pt nanoparticles and ROS generation process upon NIR light irradiation. Image reproduced with permission, from Ref Yang et al. (2017). **(F)** Schematic diagram of the process that AuNS nano-platform carries Dox enters into tumor cells by endocytosis and targets mitochondria to combine chemotherapy and PTT. Image reproduced with permission, from Ref Chen et al. (2017).

As one of the most widely studied mitochondria-targeting small molecules (*Dequalinium (DQA)*), DQA forms vesicle-like aggregates with a diameter of 70–700 nm in an aqueous solution due to the molecule’s amphiphilic nature. The resulting nanoparticles are termed DQAsomes. DQAsomes are widely used as carriers to deliver nucleic acids and cytotoxic drugs to mitochondria (Figure 7A). As a mitochondria-targeting molecule, DQA has certain selective cytotoxicity to cancer cells due to its activity of interrupting mitochondrial membrane potential, inducing ROS production and inhibiting ATP synthesis (Galeano et al., 2005). Due to the low endosomal escape ability and transfection efficiency, the potential of DQAsomes in transfection and mitochondria-targeting drug delivery is limited (Weissig et al., 1998). However, more mitochondria-targeting nanomaterials based on DQA-liposomes have recently been identified (D'Souza et al., 2003). DQAsomes may be described as the prototype of mitochondria-targeting nanocarrier systems. Since the first biological application in 1998, DQAsomes have realized the transfer of small molecule drugs and nucleic acids to mitochondria in living cells (Volkmar, 2015).

#### Inorganic Nanoparticles

With the gradual deepening of scientific research, inorganic nanomaterials have become more and more extensive in the field of biomedicine no matter from the initial chiral inorganic nanoparticles or later enriching their properties with polymer modification or self-assembly (Cumbal et al., 2003; Nie et al., 2010; Xia et al., 2011). The use of inorganic nanoparticle materials for therapeutic purposes, imaging and drug delivery have been widely reported. Compared with organic nanoparticle materials, inorganic nanoparticle materials have the advantages of hydrophilicity, low toxicity and outstanding metabolic stability (Xu et al., 2019). Recently developed mitochondria-targeting inorganic nanomaterials include graphene oxide (GO), carbon quantum dots (CQDs), lipid membrane-coated silica-carbon (LSC) and metal nanoparticles.

##### Carbon Quantum Dot (CQDs)

Due to the stable fluorescence and extremely low cytotoxicity, carbon quantum dots (CQDs) have been used as fluorescent probes in the fields of bioimaging, biomarking and biosensing (Zheng et al., 2018) (Figure 7B). Hua et al. prepared a novel fluorescent CQD with an intrinsic mitochondrial targeting capacity through one-step hydrothermal treatment without further addition of other mitochondrial ligands. The as-synthesized CQD were employed for both mitochondrial imaging and mitochondria-targeting photodynamic cancer treatment (Hua et al., 2017).

##### Graphene Oxide (GO)

Graphene oxide (GO) features high stability and large specific surface area and can bind to mitochondrial therapeutic drugs through π-π accumulation and hydrophobic interaction (Figure 7C). It has been reported that single-walled carbon nanotubes (SWNTs) can selectively target mitochondria after functionalization due to mitochondrial transmembrane potential (Zhou et al., 2010). Similar to SWNTs, GO can also target mitochondria based-on the same mechanism. Due to this intrinsic property, Wei et al. developed a photodynamic therapy (PDT) system that uses GO as a carrier to achieve drug delivery and on/off phototoxicity with mitochondrial targeting and attacking ability (Wei et al., 2016).

##### Lipid Membrane-Coated Silica-Carbon (LSC)

Wang et al. reported that the lipid membrane-coated silica-carbon (LSC) hybrid nanoparticle with the pyruvate groups can target mitochondria. This LSC nanoparticles had good bioavailability, strong optical absorption in the NIR region, good photodynamic ability and excellent PTT effect. Upon near-infrared (NIR) laser treatment, the LSC nanoparticles are able to promote mitochondrial ROS production. Experimental results further demonstrated that this novel LSC nanoparticles effectively inhibited the growth of multi-drug-resistant tumors without significant systemic toxicity (Wang et al., 2018) (Figure 7D).

##### Metal Nanoparticles

Among the metal nanoparticles, gold nanoparticles (AuNPs) are the most widely used metal nanoparticle in mitochondrial research (Costa et al., 2010; Giljohann et al., 2010; Ngwa et al., 2011). Marrache et al. co-conjugated AuNPs with TPP^+^ and the energy blocker 3-bromopyruate (3-BP) to enhance its ability to target mitochondria and inhibit the metabolic ability of cancer cells at the same time (Marrache and Dhar, 2015). Lin et al. designed a metal nanoparticle Au@Pt where Pt formed a shell over the Au core, the nanoparticle was further functionalized with folic acid, TPP^+^ and a photosensitizer, which exhibited enhanced PDT and PTT efficacy in cancer treatment (Yang et al., 2017) (Figure 7E). Chen et al. designed a nano-platform named AuNS-pep/Dox@HA based gold nanostar (AuNS), the mitochondrial targeting was achieved by the conjugation of cationic peptide R_8_ and mitochondria-targeting peptide TPP-KLA to the AuNS surface. This nano-platform combined chemotherapy (Dox) and PTT, and exhibited strong tumor growth inhibition both *in vitro* and *in vivo* (Chen et al., 2017) (Figure 7F). By using the specific reaction between 1,3-cyclohexanedione (CHD) and the sulfenic acids from oxidized proteins in tumor mitochondria, Ding et al. successfully enhanced the tumor accumulation and retention of AuNPs in cancer cells, greatly improving the sensitivity of X-ray computed tomography (CT) imaging and the radiotherapy effect of live mouse tumors. (Ding et al., 2020).

## Mitochondrial Targeting Strategies

**FIGURE 8:**
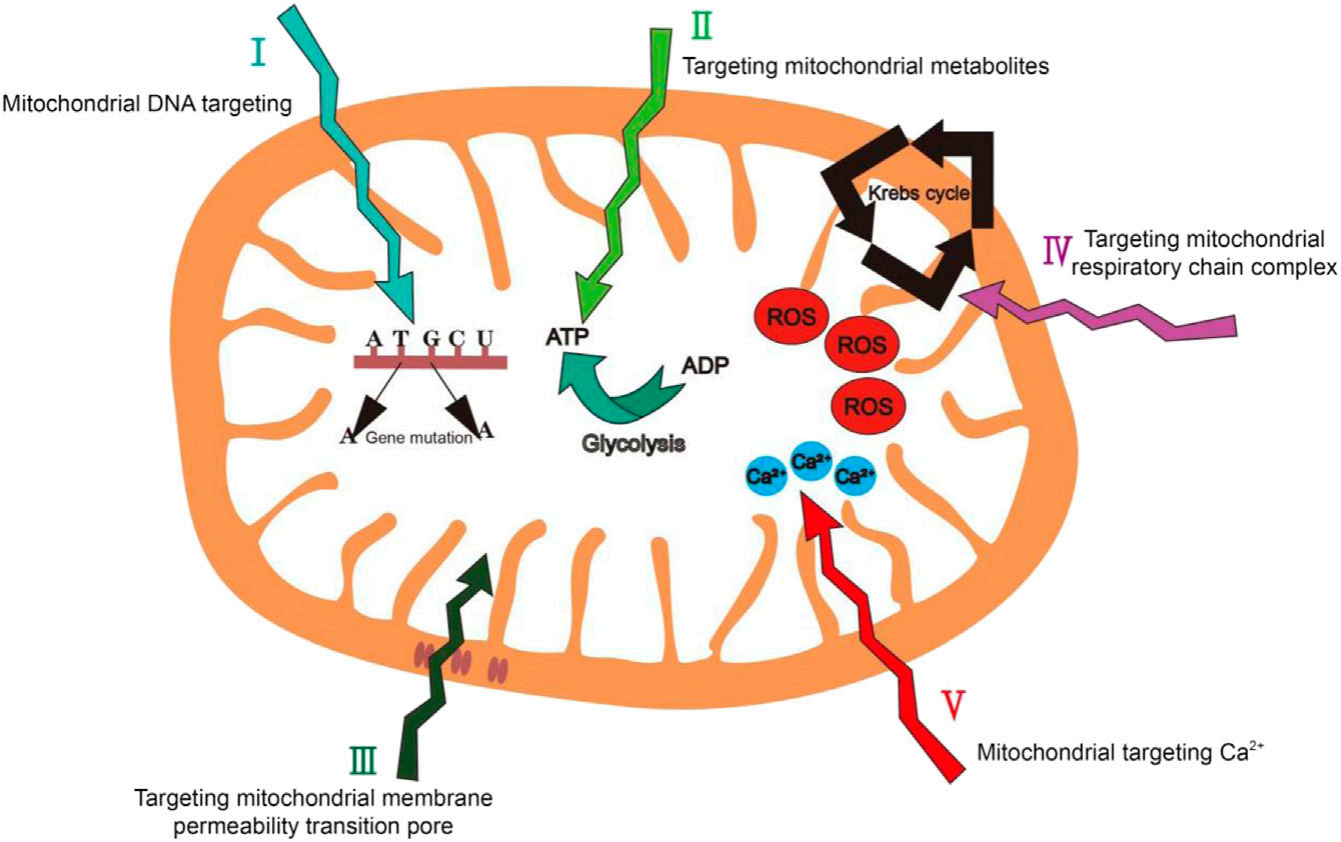
Summary of mitochondrial targeting strategies we have proposed in this review.

Based on the structure and functional characteristics of mitochondria, in addition to the well-developed targeting strategies (i.e. targeting mitochondrial membrane potential and mitochondrial membrane proteins), here we propose five promising mitochondrial biomarkers which have not been fully explored yet: 1) mitochondrial genetic information; 2) mitochondrial metabolites; 3) the mitochondrial membrane permeability transition pore 4) the mitochondrial respiratory-chain complex and 5) mitochondrial Ca^2+^ (Figure 8).

### Mitochondrial DNA

Mitochondrial DNA (mtDNA) has 16.5 kb base pairs and encodes 13 complexes. There is only one type of mtDNA in an organism. However, mtDNA lacks the protection of histidine protein, has weak repairability and is vulnerable to damage. In tumor cells, there are a large number of mtDNA mutations (Fliss et al., 2000). The base T to A or G to A is the most mutation type, and the induction of ROS may also be related to this mutation. The accumulation of mitochondrial DNA mutations leads to serious, currently incurable diseases. Therefore, it can be used as a mitochondrial biomarker in tumor cells (Gao et al., 2019).

### Mitochondrial Metabolites

Mitochondria use glycolysis to maintain cell proliferation and ATP level (Jr and Thompson, 2010). The level of cellular ATP is highly sensitive to external environmental stimuli, including hypoxia, hormones, nutrients and cytotoxic agents (Ashcroft and Gribble, 1998). In normal cells, OXPHOS is the main source of ATP. Nicotinamide adenine dinucleotide (NADH) pyruvate that is produced by oxidative glycolysis increases ATP production along the electron transfer chain through the IMM. However, during the process of tumor cell formation, glycolysis and ATP production as well as the activity of certain enzymes has changed, making tumor cells rely more on glycolysis to meet their energy needs. The changes in tumor cell metabolism provide opportunities targeting tumor cell mitochondria (Tragni et al., 2021).

### Mitochondrial Membrane Permeability Transition Pore (MPTP)

The mitochondrial membrane permeability transition pore (MPTP) a protein which is the structural basis of mitochondrial permeability transformation function. MPTP is very sensitive to the changes of the concentrations of multiple cellular ions, especially for the intracellular signal transduction system. Excessive influx of Ca^2+^ ions, oxidation of mitochondrial glutathione and increased levels of ROS will cause the continuous opening of membrane channel pores, resulting in the production of cytochrome c and the attenuation of mitochondrial membrane potential (Saotome et al., 2005). Therefore, MPTP has a very important effect in cell survival and apoptosis. The turning on of MPTP can cause mitochondria to depolarize, and abnormal MPTP turning on will result in significant change of the mitochondrial membrane potential, causing mitochondria to secrete apoptosis factors into the cytoplasm leading to cell death. The difference in MPTP between tumor cells and normal cells makes MPTP become an effective therapeutic target for the treatment of cancers (Berridge et al., 2009).

### Mitochondrial Respiratory Chain Complex

The mitochondrial respiratory chain is composed of five complexes and is located on the IMM (Rustin et al., 1997). In tumor cells, the electron transport chain activity is 20–30% lower than in normal cells (Galli et al., 2003). This is because the activity of superoxide dismutase 1 will decrease with the gradual development of tumor, while the activity of nitric oxide synthase will increase significantly, which further result in disordered level of OXPHOS in tumor cells (Sanchez-Pino et al., 2007). Therefore, the lower electron transport chain activity is usually considered as a biomarker of tumors. The mitochondria-targeting groups that are designed based on the disordered level of mitochondrial respiratory chain complex will also have an excellent targeting ability for tumor cell mitochondria.

### Mitochondrial Ca^2+^

Mitochondria play an important regulatory role in Ca^2+^ ion signals and Ca^2+^ ions have key physiological effects in the process of cell energy metabolism and signal transmission. Its overload can lead to a variety of pathological conditions, including neuronal apoptosis and death in nervous system diseases (Li et al., 2014). Therefore, targeting Ca^2+^ inside mitochondria to treat related diseases, most of which are nervous system diseases, has emerged as a promising mitochondrial targeting strategy (Waldeck-Weiermair et al., 2019).

## Summary and Outlook

In this review, we summarize and highlight the current advances in mitochondria-targeting molecules and materials (Table 1). With the discovery of more and more mitochondrial-related diseases, disease treatments that target mitochondria have received increasing attentions, which also indicates the urgency and broad prospect of the research and development of mitochondria-targeting agents. Different diseases and cellular environments have specific requirements for mitochondria-targeting agents. To be more specific, for the treatment of mitochondrial disease, the toxicity of the targeting agents needs to be reduced as much as possible. On the other hand, in order to kill harmful cells such as tumor cells, the cytotoxicity of these molecules may have a beneficial effect. In addition, mitochondria-targeting agents have also been widely used in mitochondrial imaging and monitoring, which will facilitate the physiological studies of mitochondria and generate novel mitochondrial theragnostic methods. Therefore, a variety of targeting agents is needed to meet the requirements of different situations. However, based on the current research progress of mitochondria-targeting agents, this goal has not been fully achieved.

**TABLE 1:** Summary of different types of mitochondria-targeting molecule and the corresponding targeting mechanisms and biological applications.

Targeted molecular types		Targeting mechanism		Ref.
Small molecules	TPP+	Mitochondrial membrane potential	Detection of mitochondria with fluorescent carbon dot	Wang et al. (2014)
	DQA	Mitochondrial membrane potential	Delivering drugs to breast cancer cells	Song et al. (2015)
	F16	Mitochondrial membrane potential	Treatment of cancer in combination with BODIPY	He et al. (2015)
	Guanidine/Biguanidine	Mitochondrial membrane potential	Can selectively kill prostate cancer cells	Dyshlovoy et al. (2020)
	Rhodamine	Mitochondrial membrane potential	Has good anticancer activity	Antonenko et al. (2011)
Transition metal complex	CTB	Link targeted small molecule TPP^+^	Can target mitochondria in drug-resistant tumor cells and overcome resistance to cisplatin	Zhou et al. (2014)
	Gold-1a	Can specifically target Hsp60 targets on mitochondria	Has good anticancer activity	Hu et al. (2016)
	BODIPY-Pt	Mitochondrial membrane potential	Can inhibit the proliferation of human cervical cancer and human breast cancer cells	Sun et al. (2015)
	RuL1	Mitochondrial membrane potential	Two-photon photodynamic anticancer drug for mitochondrial target	Liu et al. (2015)
	Cyolometalated Ir(III) complex	Mitochondrial membrane potential	As anticancer drug by targeting mitochondria	Cao et al. (2017)
	Complex 3	Mitochondrial membrane potential	Has good anticancer activity	Qin et al. (2017)
Bioactive molecule	MPPs	Mitochondrial membrane potential and lipotropism	Mitochondrial transport vector	Yousif et al. (2010)
	SS peptides	Unclear	Used to protect mitochondrial crista	Birk et al. (2013)
	MTSs	Recognizing specific receptors on mitochondria	Carrying medicine for treating tumor	Mossalam et al. (2012)
	CRPs	Recognizing specific receptors on mitochondria	Promote ATP production	Kam et al. (2019)
	ER signal peptide	Unclear	Temporary non-biological application	Pfeiffer et al. (2013)
	mRNA	Unclear	Temporary non-biological application	Michaud et al. (2014)
Nanomaterials	Polymeric/polymer-coated nanoparticles and micelles	Binding to molecules with targeting capability	Can deliver drug, treat cancer and tumor	Zheng et al. (2012); Chen et al. (2015); Ma et al. (2018)
	DQAsomes and DQA-liposomes	Mitochondrial membrane potential and lipotropism	Transfer of small molecule drugs and nucleic acids to the mitochondria of living cells	Volkmar (2015)
	Inorganic nanoparticles	Binding to molecules with targeting capability	For mitochondrial image and mitochondrial targeted photodynamic cancer treatment	Hua et al. (2017)

Mitochondria-targeting small molecules are widely used in various applications. Most of these mitochondria-targeting molecules can be directly conjugated with drugs, which have the advantage of enabling relatively accurate targeted delivery of drugs to the mitochondria. Among all the mitochondria-targeting molecules, TPP^+^ is the most commonly used molecule due to the high efficiency, flexible conjugation strategy and low cost. While Rh19, as the successor, holds several advantages over TPP^+^, although the applications of Rh19 in biological studies still need to be further explored. However, the conjugation of such mitochondria-targeting molecules and drugs generally has the disadvantage of poor water solubility, and some mitochondria-targeting ligands have significant cytotoxicity. Notably, due to the bulky size of those molecules, direct conjugation of them to drugs may affect the activity and therapeutic effects of the conjugated drugs. Therefore, mitochondria-targeting small molecules that have low cytotoxicity, high water solubility and relatively small size should be the future development direction.

Mitochondria-targeting biomolecules are a kind of targeting groups with good development prospects at present. Most of these mitochondria-targeting macromolecules are amino acid-based polypeptides. Compared with the mitochondria-targeting small molecules, biomolecules have excellent specificity against mitochondria to a great extent without causing adverse effects on other organelles or cells. This kind of targeting agent has little or even negligible toxicity to cells. However, the exact targeting mechanism of many mitochondria-targeting biomolecules that have been identified and applied is still unclear. Thus, the development direction of such mitochondria-targeting groups is not only to find new substances that can target mitochondria, but also to explore their targeting mechanism. We believe that the discovery of the targeting mechanism of these targeting groups will greatly promote their development and clinical applications.

Mitochondria-targeting nanomaterials can be used as a platform or carrier to shuttle a drug payload to the organelle, so they should be considered the most urgently needed targeting group for development in clinical application. However, due to the uncertainty of their *in vivo* distribution, immunogenicity and excretion, the clinical application of these nanomedicines is still lagging behind. Mitochondria-targeting nanomaterials should continue to develop toward multi-function, improve the efficiency of treatment. However, many of them are currently only limited to laboratories or research institutes. Future development, such as the enhancement of their blood-brain barrier permeability will hold great potential in the treatment of neurodegenerative diseases which are widely linked with mitochondrial dysfunction.

In summary, research on mitochondria-targeting moieties still has a long way to go. It not only needs to meet the requirements of scientific research, but more importantly, it needs to be applied clinically to meet various requirements for the treatment of mitochondrial diseases and solve more mitochondrial medical problems.
